# Dicer-independent miR-451a is upregulated in periodontitis and potentiates inflammation by impairing macrophage polarization and SOCS activity

**DOI:** 10.1007/s00011-026-02381-9

**Published:** 2026-10-06

**Authors:** Raza Ali Naqvi, Jack Maddalozzo, Kreena Amin, Kristelle Capistrano, Araceli Valverde, Rani Rahat, Hasan Siddiqui, Wei Li, Christine D. Wu, Shaoping Zhang, Mary Pettiette, Antonio Moretti, Gatikrushna Singh, Salvador Nares, Afsar Naqvi

**Affiliations:** 1 Department of Periodontics, College of Dentistry, University of Illinois Chicago, Room 461, Chicago, IL 60612 USA; 2 Department of Pediatric Dentistry, College of Dentistry, University of Illinois Chicago, Chicago, IL 60612 USA; 3 Department of Periodontics, College of Dentistry and Dental Clinics, University of Iowa, Iowa City, IA 52242 USA; 4 Department of Endodontics, School of Dentistry, Virginia Commonwealth University, Richmond, VA 23298 USA; 5 Department of Periodontology, School of Dentistry, University of North Carolina at Chapel Hill, Chapel Hill, NC 27599 USA; 6 Department of Neurosurgery, University of Minnesota, Minneapolis, MN 55455 USA; 7 Mucosal Immunology Lab, Department of Microbiology and Immunology, College of Medicine, University of Illinois Chicago, Room 561 B, Chicago, IL 60612 USA

**Keywords:** Periodontal disease, Split-mouth, Macrophage, Noncanonical microRNAs, Polarization

## Abstract

**Background:**

Periodontitis (PD) is characterized by localized inflammatory tissue destruction, yet the lesion-associated microRNA (miRNA)networks that regulate host immune responses remain incompletely defined. In this study, we aimed to identify and characterize dysregulated gingivalmiRNAs in PD that modulate inflammatory responses.

**Methods:**

Using a split-mouth design, miRNA expression was profiled in gingival biopsies fromperiodontal lesions and clinically healthy sites. Differentially expressed miRNAs were identified by microarray analysis, and selected candidates werevalidated by RT-qPCR. The responses of candidate miRNAs to periodontal bacterial challenge were evaluated, followed by functional studies ofmacrophage polarization, phagocytosis, inflammatory cytokine production, and target-gene regulation.

**Results:**

Microarray analysis identified 48differentially expressed miRNAs, including several upregulated non-canonical miRNAs. Among these, the Dicer-independent miRNAs miR-451a andmiR-1228 showed dose- and time-dependent induction following periodontal bacterial challenge. miR-451a, but not miR-1228, promoted a pro-inflammatory M1-like macrophage phenotype, characterized by increased HLA-DR and CD32 and reduced CD206 and CD163 expression. miR-451adirectly targeted multiple genes associated with M2 macrophage polarization, and its expression in inflamed gingival tissues inversely correlated with M2markers. miR-451a overexpression also reduced macrophage phagocytosis of E. coli and P. gingivalis while increasing pro-inflammatory cytokineproduction. Mechanistically, miR-451a directly suppressed SOCS3 and SOCS5, key negative regulators of JAK/STAT signaling, in TLR4-stimulated cells.The antagonistic expression patterns of miR-451a, SOCS3, and SOCS5 in PD further supported the functional relevance of this regulatory axis.

**Conclusion:**

miR-451a is a pathogen-responsive, PD-associated miRNA that promotes pro-inflammatory macrophage polarization, impairs bacterialphagocytosis, and amplifies inflammation through suppression of SOCS-mediated immune regulation. These findings identify the miR-451a–SOCS3/SOCS5 axis as a potential mechanism linking bacterial challenge to dysregulated macrophage responses in PD.

**Supplementary Information:**

The online version contains supplementary material available at https://doi.org/10.1007/s00011-026-02381-9.

## Introduction

Periodontal disease (PD) is an inflammatory condition of tooth-supporting tissues initiated by microbial dysbiosis and sustained by an overt host immune-inflammatory response [1–3]. Accumulating evidence, including work from our laboratory, shows that recruitment and persistence of pro-inflammatory immune cells in gingiva are closely linked to disease onset and progression. Diseased gingiva consistently contains increased numbers of myeloid cells, T cells, and B cells compared with healthy tissue, along with elevated levels of immunoglobulins, cytokines, and other inflammatory mediators [4–7].

Macrophages (Mφ) are key myeloid cells in oral mucosa and are actively recruited to periodontal lesions during disease progression. Their roles in PD pathogenesis and resolution are well established [6, 8, 9]. Mφ play important role in the early host response to periodontal infection by recognizing microbe-associated signals through PRRs, including TLR2 and TLR4, and coordinating immune responses that contribute to effective pathogen clearance [10, 11]. In PD, Mφ exist along a continuum of activation states ranging from pro-inflammatory M1-like to pro-reparative M2-like phenotypes. Our group and others have demonstrated that PD subjects display an increased M1/M2 ratio that declines after non-surgical periodontal therapy [6, 12, 13], supporting their role in both inflammation and resolution. Persistent M1-like activation promotes pro-inflammatory cytokine release and metalloproteinase activity, driving collagen destruction and alveolar bone loss [14]. In contrast, M2 Mφ produce anti-inflammatory mediators such as IL-10 and TGF-β, suppress osteolysis, and promote tissue repair [15, 16]. In murine periodontal tissues, adoptive transfer of M2 Mφ increased Treg abundance and reduced osteoclast activity, a central mediator of alveolar bone loss [17]. Together, these findings underscore the importance of identifying molecular regulators of Mφ polarization.

MicroRNAs (miRNAs) are approximately 22-nucleotide non-coding RNAs that regulate gene expression post-transcriptionally [18–20]. In the canonical pathway, primary miRNAs are transcribed and processed in the nucleus before being exported to the cytoplasm, where precursor miRNAs are cleaved by Dicer to generate mature miRNA duplexes. Some miRNAs are generated through non-canonical pathways, including mirtrons, Dicer-independent miRNAs, and species derived from tRNAs or snoRNAs [21–24]. A well-known example is miR-451a, which bypasses Dicer and is processed directly through AGO2 after DROSHA-DGCR8 cleavage and nuclear export [21, 22]. Regardless of biogenesis route, mature miRNAs regulate target transcripts through the same core AGO/RISC-mediated mechanisms.

Although the role of miRNAs in immune disorders is increasingly recognized, their regulatory functions and therapeutic potential in PD remain unclear. Our group and other reports have demonstrated differential miRNA expression in inflamed gingiva and tooth pulps [25–28]. Moreover, the functional roles and mechanistic impact of non-canonical miRNAs in PD have never been investigated. This study investigated the functional roles of the non-canonical miRNAs miR-451a and miR-1228 in macrophage polarization and identified molecular mechanisms through which these miRNAs regulate anti-inflammatory pathways involved in periodontal immune responses.

## Materials and methods

### Study population and sample collection

This study protocol was approved by the institutional review board of the University of North Carolina at Chapel Hill (#13–1279). Systemically healthy adults (N = 9) aged 18–70 years who presented to the Postgraduate Periodontics Clinic, Adams School of Dentistry, UNC Chapel Hill, between March 2013 and May 2015 were recruited. Participants were excluded if they had uncontrolled systemic conditions (e.g., hypertension, heart disease, bleeding disorders), were pregnant, had diabetes, or were current smokers. Periodontally healthy sites had probing depths (PPD) ≤ 3 mm, no bleeding on probing, no clinical attachment loss (CAL), no radiographic alveolar bone loss, and ≥ 4 mm of attached keratinized gingiva. Periodontally diseased sites met criteria for stage III, grade B disease, including ≥ 4 teeth with PPD ≥6 mm, CAL ≥5 mm, and radiographic bone loss extending to the middle third of the root and beyond, with ≥ 4 mm attached keratinized gingiva, as previously described [28, 29]. Table 1 summarizes demographic and clinical data of the cohort.

**Table 1:** Subject demographics and clinical parameters

Age (mean± SD) (years)	56.78 ± 11.28		
Gender (M/F)	5/4		
Clinical site variables	Periodontitis site (n = 9)	Non-periodontitis site (n = 9)	*p*-value*
PPD (mean± SD) (mm)	7.33 ± 1.85	2.56 ± 0.73	< 0.001
CAL (mean± SD) (mm)	6.67 ±1.80	2.33 ± 0.87	< 0.001

^*^Paired t-test

Participants with PD had 1–2 mm^2^ of gingiva removed from the tooth with the deepest probing site when undergoing surgical treatment. Additionally, a second sample of identical size was collected from a clinically healthy site elsewhere in the oral cavity during the same surgical intervention. The harvested tissue samples were placed in sterile round bottom polypropylene tubes and immediately immersed in 1 mL RNAlater (Applied Biosystems/Ambion, Austin, TX, USA) solution and incubated at 4 °C for at least 24 h. The processed samples were then transferred to − 80 °C for long-term storage.

### MicroRNA microarray profiling

Tissue samples were lysed using a TissueLyzer (Qiagen, Germantown, MD, USA), and total RNA was isolated using the miRNeasy Kit (Qiagen), as previously described [29, 30]. The miRNA microarray assay was performed by LC Sciences (Houston, TX, USA). For miRNA labeling, a strand-labeling reaction was performed using 1 μg of total RNA, control oligonucleotides (Integrated DNA Technologies, Coralville, IA, USA), App-Cap3, T4 RNA ligase (Rnl2tr-K227Q; New England BioLabs, Ipswich, MA, USA), T4 RNA ligase buffer containing PEG8000, and an RNase inhibitor (Promega, Madison, WI, USA). The reaction mixture was incubated at 16 °C for 16 h and then diluted 1:1 with hybridization buffer. A subsequent dye-labeling step was performed using a tag sequence designed to capture the fluorescent dye. Hybridization was performed at 40 °C for 16 h on a μParaflo microfluidic chip using a microcirculation pump. The detection probes contained chemically modified nucleotide sequences complementary to the target miRNAs identified in miRBase, as well as control and custom-defined RNA sequences. The probes also contained a polyethylene glycol spacer to spatially separate the target-binding region from the substrate. Detection probes were generated by in situ synthesis using photogenerated reagent (PGR) chemistry and chemically modified to optimize melting temperatures and hybridization efficiency. Hybridization was performed in 100 μL of 6× SSPE buffer (0.90 M NaCl, 60 mM Na₂HPO₄, and 6 mM EDTA; pH 6.8) containing 25% formamide. Following RNA hybridization, a Cy3-conjugated dye was circulated through the microfluidic chip for fluorescence detection. Fluorescence images were acquired using a GenePix 4000B laser scanner (Molecular Devices, San Jose, CA, USA) and analyzed using Array-Pro image analysis software (Media Cybernetics, Rockville, MD, USA) to generate quantitative signal data. Background signals were subtracted, and the resulting fluorescence signals were normalized using a LOWESS (locally weighted scatterplot smoothing) method. The miRNA microarray data were deposited in the Gene Expression Omnibus (GEO) repository in accordance with MIAME guidelines and are available under accession number GSE339403.

### Total RNA isolation and quantitative RT-PCR

Total RNA was isolated using the miRNeasy Mini Kit (Qiagen, Hilden, Germany) following the manufacturer's protocol. A total of 100 ng RNA was reverse transcribed. Reverse-transcription reactions were performed using a PCR master mix containing SYBR Green (Roche, Indianapolis, IN, USA) and the reactions were run using either a universal primer with a miRNA-specific primer (Qiagen) or gene-specific primers (Sigma). RNU6B was used as an endogenous control. Relative miRNA expression was calculated using the comparative ΔΔCt method, and normalized expression values were presented as mean ± SD.

### Transient miRNA transfection

Transient transfections were carried out using Lipofectamine 2000 reagent (Life Technologies, San Diego, CA, USA) following the manufacturer instructions. To determine the efficiency of the transfection reactions, red siGLO oligos (ThermoScientific, Waltham, MA, USA) were used. Cells were then harvested for RNA isolation after 36 h. Three increasing concentrations of 10, 20, and 50 nM (to determine dose effects) of miRNA mimics were used to transfect the cells. Because robust transfection efficiency (~ 90%) was observed at 20 nM, this concentration was used for most experiments unless otherwise indicated. Controls consisted of mock transfected and control mimic (Qiagen) transfected cultures.

### Primary human macrophage differentiation and challenge with periodontal bacteria

Monocytes were isolated from freshly prepared buffy coats collected from healthy donors (n ≥ 4, Sylvan N. Goldman Oklahoma Blood Institute, Oklahoma City, OK, USA) by density gradient centrifugation and CD14+ cells were sorted by magnetic bead sorting as previously described [31–33]. For Mφ differentiation, monocytes were plated at 2 × 10^6^/ml in DMEM supplemented with penicillin (100 U/mL) and streptomycin (100 μg/mL). After 2 h, the medium was replaced with medium containing 10% FBS (Life Technologies, Carlsbad, CA, USA), and rhM-CSF (50 ng/mL; Peprotech, Rocky Hill, NJ, USA). Cells were stimulated with live *Aggregatibacter actinomycetemcomitans* (*Aa*, strain Y4, serotype B) or *Porphyromonas gingivalis* (*Pg*, strain W83) at 100 MOI.

### Flow cytometry, TLR stimulation and phosphoflow

Cells were stained for different antibodies and analyzed using BD Accuri C6 flow cytometer as described before [32, 33]. For phosphoflow experiments, cells were stimulated with TLR4 (*E. coli* LPS, 50 ng/mL) stimulation and flow cytometry was performed as described previously [33]. Primary human M1 or M2-differentiated Mφ were plated in 96-well plates at 4×10^5^ cells/well and transfected with miR-451a, miR-1228 or control mimics on day 3. At day 6, cells were collected and washed in ice-cold PBS containing 1% (v/v) FBS and 0.08% sodium azide. Initial gating was performed on forward scatter (FSC) and side scatter (SSC) to remove debris and select the main leukocyte population; the monocyte gate accounted for approximately 60% of total events. Doublets/cell aggregates were excluded using FSC vs SSC and SSC vs pulse width gating. For phenotypic analysis, M1 and M2 Mφ were stained with fluorophore-conjugated antibodies (FITC, PE, and APC) against polarization markers. Antibodies included CD32-PE and HLA-DR-FITC (M1-associated markers) and CD163-APC and CD206-PE (M2-associated markers), sourced from BD Pharmingen (San Diego, CA, USA) and/or BioLegend (San Diego, CA, USA). Unstained and isotype controls (BD Pharmingen) were included to set gates and assess nonspecific binding. Data were acquired on a BD Accuri™ C6 flow cytometer (BD Biosciences, San Jose, CA, USA) and analyzed using FlowJo (Tree Star, Ashland, OR, USA). Final gates were defined using FSC/SSC characteristics to retain larger, more granular cells while excluding small, low-FSC/low-SSC debris (typically appearing in the lower-left region of the dot plot).

To evaluate SOCS expression following TLR4 stimulation, miRNA-transfected Mφ were cultured in RPMI-1640 and stimulated overnight (18 h) with the TLR4 agonist *E. coli* lipopolysaccharide (LPS; 50 ng/mL; Human Toll-like Receptor kit, InvivoGen). Following stimulation, cells were harvested, washed in RPMI containing 1% BSA, and fixed with Cytofix buffer (BD Biosciences) at 4 °C for 30 min. Cells were then permeabilized according to the manufacturer’s instructions, washed twice, and stained with 5 μl FITC-conjugated anti-SOCS3, anti-SOCS5 and anti-SOCS6 (BioLegend). Protein levels were quantified by flow cytometry, and the frequency of SOCS+ cells was determined relative to control mimics.

### Phagocytosis assays

Cells transfected with miRNA or control mimics were assayed for phagocytosis using rhodamine labeled *E. coli* and *P. gingivalis*. Human primary Mφ were plated in 96-well plates at 4 × 10^5^ cells/well and transfected with miR-451a, miR-1228 or control mimics (25 nM final concentration). Phagocytosis was assessed 36 h post-transfection using rhodamine conjugated *E. coli* bioparticles (Invitrogen, Carlsbad, CA, USA), following the manufacturer’s protocol. Briefly, labeled *E. coli* bioparticles or *P. gingivalis* were resuspended in Live Imaging Buffer (Life Technologies) to a final concentration of 1 mg/mL, homogenized by sonication for 2 min, and then diluted in culture medium. Mφ were incubated with the labeled bioparticles for 2 h at 37 °C, washed three times with PBS, and fixed with 4% paraformaldehyde. Random fields were imaged for each donor (n = 4) using an EVOS microscopy system (Thermo Fisher Scientific, Grand Island, NY, USA). In parallel, uptake of rhodamine-labeled particles was quantified by flow cytometry as previously described [32].

### Cell viability assay

Cell viability was determined by use of the CellTiter 96 AQueous Cell Proliferation Assay Kit (Promega). In brief, Mφ were plated at 400,000/well in 96-well plates and transfected as described above and assays were performed after 36 h, according to the manufacturer's instructions [6].

### Bioinformatic target prediction, cloning of gene 3' UTRs, and dual luciferase assays

Gene targets were identified using miRWalk (http://mirwalk.umm.uni-heidelberg.de/). For miRNA pathway analysis, DIANA tool mirPath v.3 (https://dianalab.e-ce.uth.gr/html/mirpathv3) was used to identify significantly overrepresented pathways and functional categories. Cloning of predicted gene 3′ untranslated region (UTR) and dual luciferase assays were performed as previously described [31, 34]. Full-length 3′UTRs were PCR-amplified using genomic DNA isolated from monocytes as the template and cloned into the psiCHECK-2 dual-luciferase reporter vector (Promega) by ligating each 3′UTR downstream of the Renilla luciferase gene using the XhoI and NotI restriction sites in the multiple cloning site. Dual-luciferase reporter assays were carried out as previously described. HEK293 cells were plated in 96-well plates at 3 × 10^4^ cells/well in DMEM supplemented with 10% fetal bovine serum. Cells were co-transfected using Lipofectamine 2000 (Invitrogen; 0.5 µL/well) with 120 ng of the psiCHECK-2 dual-luciferase reporter plasmid containing a target gene 3'UTR harboring the miRNA binding site, together with synthetic hsa-miR-451a and miR-1228 mimics at different doses. A control mimic was included at 50 nM (Qiagen). After 36 h post-transfection, cells were lysed using Passive Lysis Buffer (Promega), and Renilla and firefly luciferase activities were quantified using a Victor™ X5 multilabel plate reader (PerkinElmer 2030; PerkinElmer Health Sciences Inc., Shelton, CT, USA).

#### Global pathway analysis

miRNA functional enrichment analysis was performed using DIANA-miRPath v3.0 to investigate the biological pathways and functional categories potentially regulated by the selected miRNAs. The submitted miRNA list was analyzed using the annotation resources available in miRPath v3 (https://dianalab.e-ce.uth.gr/html/mirpathv3), including Gene Ontology (GO) annotations. For target identification, TarBase was used as the reference database, thereby restricting the analysis to experimentally validated miRNA-target interactions.

Enrichment analysis was conducted within the miRPath v3 platform by mapping validated miRNA target genes to GO terms to identify significantly overrepresented functional categories. Statistical significance was assessed using the built-in miRPath v3 analytical framework, with a threshold of P < 0.05. False discovery rate (FDR) adjustment was applied to correct multiple comparisons, and only pathways or GO terms with an FDR-corrected P value < 0.05 were considered statistically significant. Significantly enriched KEGG pathways and GO annotations were retained for downstream biological interpretation of the potential functions associated with the analyzed miRNAs.

#### Cytokine analysis

Supernatants were collected from miR-451a or control mimic transfected Mφ challenged with *E. coli* at 4 and 24 h. Multiplex analysis of four different cytokines (IL-1β, IL-6, IL-8 and TNF-α) was performed using Milliplex (Millipore, Billerica, MA, USA). Data were collected on a Bio-Plex flow cytometer (Bio-Rad, Hercules, CA, USA) for analysis.

#### Statistical analysis

Data were analyzed using GraphPad Prism software (GraphPad Software, La Jolla, CA, USA). The results are presented as mean ± SEM of 3–5 independent replicates and experiments were conducted at least three times. p-values were calculated using Student’s t-test or ANOVA, and *p* < 0.05 was considered significant. For the microarray analysis, data were normalized using appropriate statistical methods, distributional assumptions were assessed prior to downstream analyses, and multiple testing correction was applied using the false discovery rate (FDR) method to control for potential false-positive findings.

## Results

### Gingival microRNA profiles reveal site-specific molecular changes associated with periodontal disease

The cohort included adults with a mean age of 56.78 ± 11.28, including 5 males and 4 females. Using a split-mouth design, we collected one gingival tissue biopsy from a periodontally diseased and non-diseased site (n = 9 per site type) from each subject. Diseased sites had greater PPD (7.33 ± 1.85 mm) than non-diseased sites (2.56 ± 0.73 mm,* p* < 0.001) (Table 1). CAL was also higher at diseased sites (6.67 ± 1.80 mm) than at non-diseased sites (2.33 ± 0.87 mm,* p* < 0.001), confirming clear separation between PD and non-diseased sites.

**Fig. 1:**
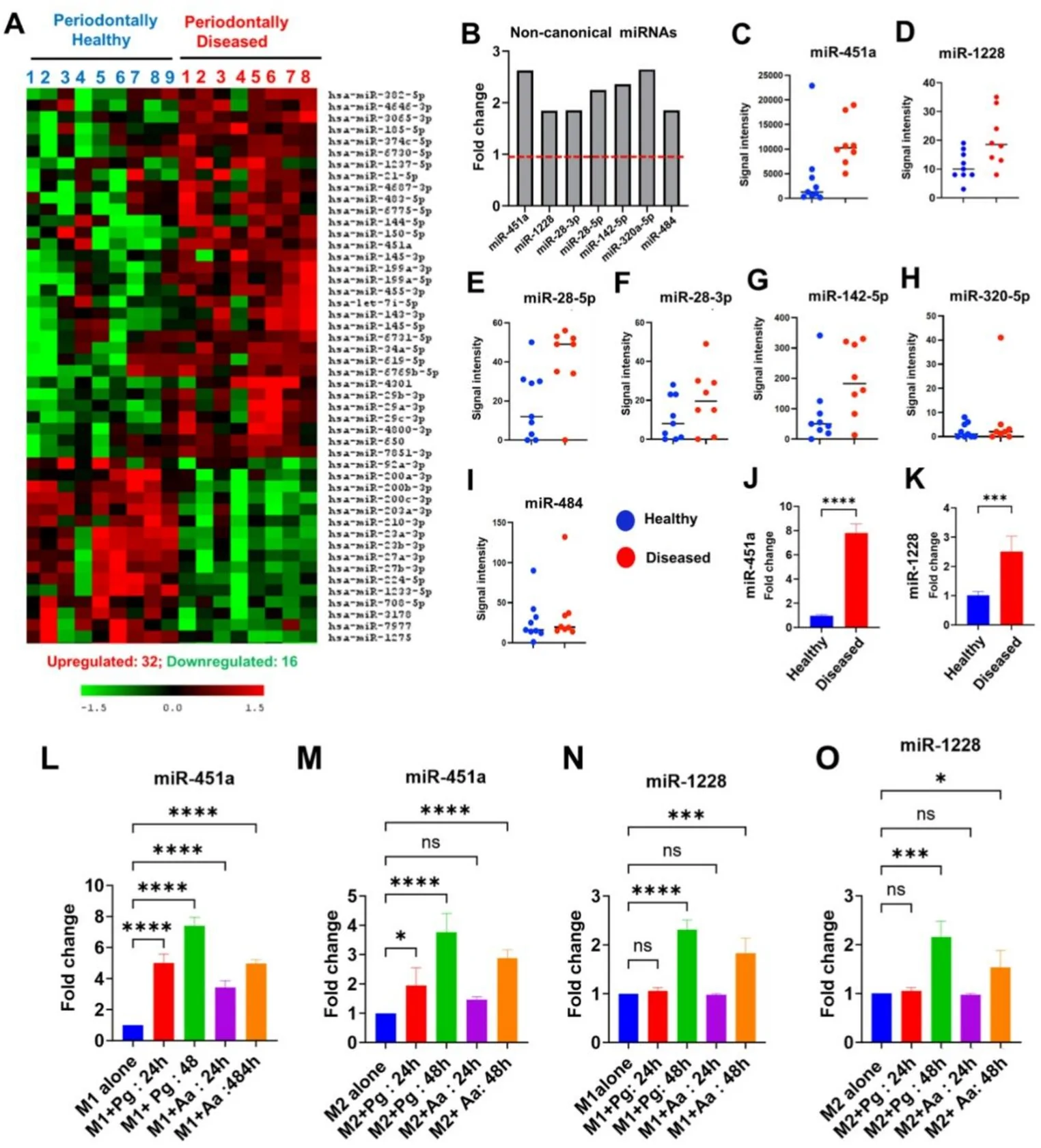
Split-mouth miRNA profiling reveals distinct expression signatures in inflamed versus healthy gingiva and identified periodontopathogen-induced Dicer-independent miRNAs.** A** Total RNA isolated from clinically healthy (n = 9) and inflamed (n = 8) gingival biopsies from a split-mouth design was subjected to global miRNA profiling using microarray analysis. Heatmaps depict significantly upregulated and downregulated miRNAs in inflamed gingiva compared with healthy sites. **B** Fold-change values for selected non-canonical miRNAs in inflamed versus healthy gingiva. **C**–**I** Average signal intensities for individual miRNAs, including **C** miR-451a, **D** miR-1228, **E** miR-28-5p, **F** miR-28-3p, **G** miR-142-5p, **H** miR-320-5p, and **I** miR-484, in healthy and inflamed gingival tissues. Validation of Dicer-independent miRNAs was performed by RT-qPCR. Relative expressions of **J** miR-451a and **K** miR-1228 in healthy versus inflamed gingiva were quantified using RNU6 as an endogenous control. Data are presented as mean ± SEM. Dicer-independent miRNAs are responsive to periodontal pathogens. Primary M1- and M2-like macrophages (Mφ) were challenged with 100 MOI of *P. gingivalis* or *A. actinomycetemcomitans* for 24 h and 48 h, and the expression of **L**, **M** miR-451a and **N**, **O** miR-1228 was quantified by RT-qPCR. RNU6 was used as endogenous control. Data are presented as mean ± SEM from four independent donors. Statistical significance was determined by ANOVA; *p* values are indicated as *p < 0.05, **p < 0.01, ***p < 0.001; ****p < 0.0001

Microarray profiling identified 48 differentially expressed miRNAs between inflamed and healthy gingiva, showing clear site-specific dysregulation. Heatmap analysis demonstrated distinct clusters of upregulated and downregulated miRNAs that separated diseased from healthy sites (Fig. 1A). Of the 48 differentially expressed miRNAs, 32 were upregulated and 16 were downregulated in inflamed gingiva. The most upregulated included miR-191a-3p, miR-150-5p, miR-451a, miR-4646-3p, miR-6851-3p, and miR-202-5p, whereas miR-23a/b, miR-27a/b, miR-224, miR-1275, and miR-1233 were among the most downregulated (Table 2).

**Table 2:** Differentially expressed miRNAs in periodontally healthy and diseased gingival biopsies

miRNA	Healthy (Mean intensity)	Disease (Mean intensity)	*P*-value	Fold change
hsa-miR-200c-3p	752	252	1.53E−05	0.34
hsa-miR-200b-3p	475	177	1.03E−04	0.37
hsa-miR-23b-3p	3648	2015	1.44E−04	0.55
hsa-miR-23a-3p	4033	2491	3.58E−04	0.62
hsa-miR-199a-3p	661	1716	3.73E−04	2.60
hsa-miR-203a-3p	2899	434	4.83E−04	0.15
hsa-miR-27b-3p	720	363	8.47E−04	0.50
hsa-miR-143-3p	202	424	4.01E−03	2.10
hsa-miR-199a-5p	320	816	4.60E−03	2.55
hsa-miR-451a	4284	11,244	5.88E−03	2.62
hsa-miR-27a-3p	1127	647	6.02E−03	0.57
hsa-miR-29a-3p	619	1165	8.55E−03	1.88
hsa-miR-145-5p	371	703	1.06E−02	1.90
hsa-miR-29c-3p	243	625	1.11E−02	2.58
hsa-miR-150-5p	193	347	1.13E−02	1.79
hsa-miR-3178	248	102	1.16E−02	0.41
hsa-miR-7977	326	175	1.48E−02	0.54
hsa-miR-1233-5p	225	23	1.75E−02	0.10
hsa-miR-29b-3p	315	768	1.81E−02	2.44
hsa-let-7i-5p	419	748	3.27E−02	1.79
hsa-miR-21-5p	3431	5245	4.76E−02	1.53
hsa-miR-148a-3p	362	808	5.49E−02	2.23
hsa-miR-140-3p	217	337	7.14E−02	1.55
hsa-miR-1246	257	342	7.92E−02	1.33
hsa-miR-223-3p	113	478	8.15E−02	4.25
hsa-miR-4530	168	266	8.33E−02	1.59
hsa-miR-205-5p	3294	1273	8.49E−02	0.39
hsa-miR-455-3p	50	91	6.29E−04	1.83
hsa-miR-3065-3p	1	10	9.02E−04	19.57
hsa-miR-224-5p	68	28	2.69E−03	0.41
hsa-miR-4301	108	290	5.46E−03	2.68
hsa-miR-708-5p	73	37	5.75E−03	0.51
hsa-miR-4800-3p	14	43	1.00E−02	3.04
hsa-miR-210-3p	19	8	1.13E−02	0.42
hsa-miR-185-5p	24	54	1.15E−02	2.22
hsa-miR-6775-5p	14	47	1.21E−02	3.32
hsa-miR-200a-3p	157	73	1.37E−02	0.47
hsa-miR-1275	138	57	1.42E−02	0.41
hsa-miR-483-5p	18	38	1.62E−02	2.11
hsa-miR-7851-3p	11	49	1.66E−02	4.58
hsa-miR-34a-5p	33	61	1.66E−02	1.84
hsa-miR-92a-3p	263	170	1.76E−02	0.65
hsa-miR-1237-5p	20	38	1.89E−02	1.95
hsa-miR-374c-5p	32	68	2.22E−02	2.12
hsa-miR-145-3p	9	47	2.43E−02	5.02
hsa-miR-144-5p	14	42	2.49E−02	2.90
hsa-miR-650	9	45	2.51E−02	4.86
hsa-miR-6769b-5p	13	24	2.56E−02	1.91
hsa-miR-4687-3p	56	124	2.70E−02	2.23
hsa-miR-6731-5p	3	9	3.52E−02	3.12
hsa-miR-382-5p	6	18	4.40E−02	3.33
hsa-miR-4646-3p	6	17	4.45E−02	2.85
hsa-miR-6730-5p	7	20	4.78E−02	2.74
hsa-miR-619-5p	19	52	4.95E−02	2.69

Mean signal intensity, fold change, and p-values (significance threshold* p* < 0.05) are shown for miRNAs significantly dysregulated between healthy and inflamed gingiva. miRNAs are ordered by ascending* p*-value

Several non-canonical miRNAs were also increased in inflamed gingiva, including miR-451a (2.6-fold), miR-1228 (1.3-fold), miR-142-5p (2.3-fold), miR-28-3p (1.8-fold), miR-28-5p (2.2-fold), miR-484 (1.3-fold), and miR-320-5p (2.6-fold) (Fig. 1B). Their average signal intensities also trended higher in inflamed tissue (Fig. 1C–I). RT-qPCR validation confirmed significantly increased miR-451a (~ 8-fold) and miR-1228 (~ 2-fold) expression in inflamed biopsies relative to clinically healthy controls (Fig. 1J and K). Together, these results show that Dicer-independent miRNAs display a distinct expression pattern in inflamed gingiva, supporting a role for site-specific miRNA dysregulation in periodontal pathogenesis.

### miR-451a and miR-1228 are responsive to periodontal bacteria

Periodontal pathogens modulate innate immune programs in Mφ, including dysregulation of noncoding RNA expression that can rapidly shape inflammatory response and resolution. To examine whether the expression of non-canonical, Dicer-independent miRNAs miR-451a and miR-1228 is responsive to periodontal bacteria, we quantified their expression in M1 and M2 Mφ challenged with 100 MOI live *Pg* and *Aa* at 24 and 48 h. In M1 macrophages, miR-451a expression increased in a time-dependent manner following challenge with either bacterium, with significant induction observed at 24 h (*Pg*: 4.98 ± 0.58 fold; *Aa*: 3.42 ± 0.40 fold) and further at 48 h (*Pg*: 7.4 ± 0.45 fold; *Aa*: 5.0 ± 0.21 fold) (n = 6/group; Fig. 1 L and M). In contrast, miR-1228 expression in M1 Mφ showed no significant change at 24 h (*Pg*: 1.05 ± 0.06 fold; *Aa*: 0.97 ± 0.02 fold) but was increased at 48 h (*Pg*: 2.31 ± 0.20 fold; *Aa*: 1.83 ± 0.30 fold) (Fig. 1 N and O). These results indicate that miR-451a is induced rapidly and remains persistently high, whereas miR-1228 shows a delayed induction profile. Together, these findings suggest that non-canonical miRNAs are responsive to periodontal pathogens.

### miR-451a regulates macrophage polarization by skewing a shift towards M1 phenotype

Macrophages are central to both the persistence and resolution of gingival inflammation, coordinating pro-inflammatory and reparative responses [6, 35]. To identify endogenous regulators of Mφ polarization, we examined the effects of the Dicer-independent miRNAs miR-451a and miR-1228. These candidates were selected based on high expression, significant differential regulation, and predicted targeting of immune and inflammatory pathways (Supplementary file 1).

**Fig. 2:**
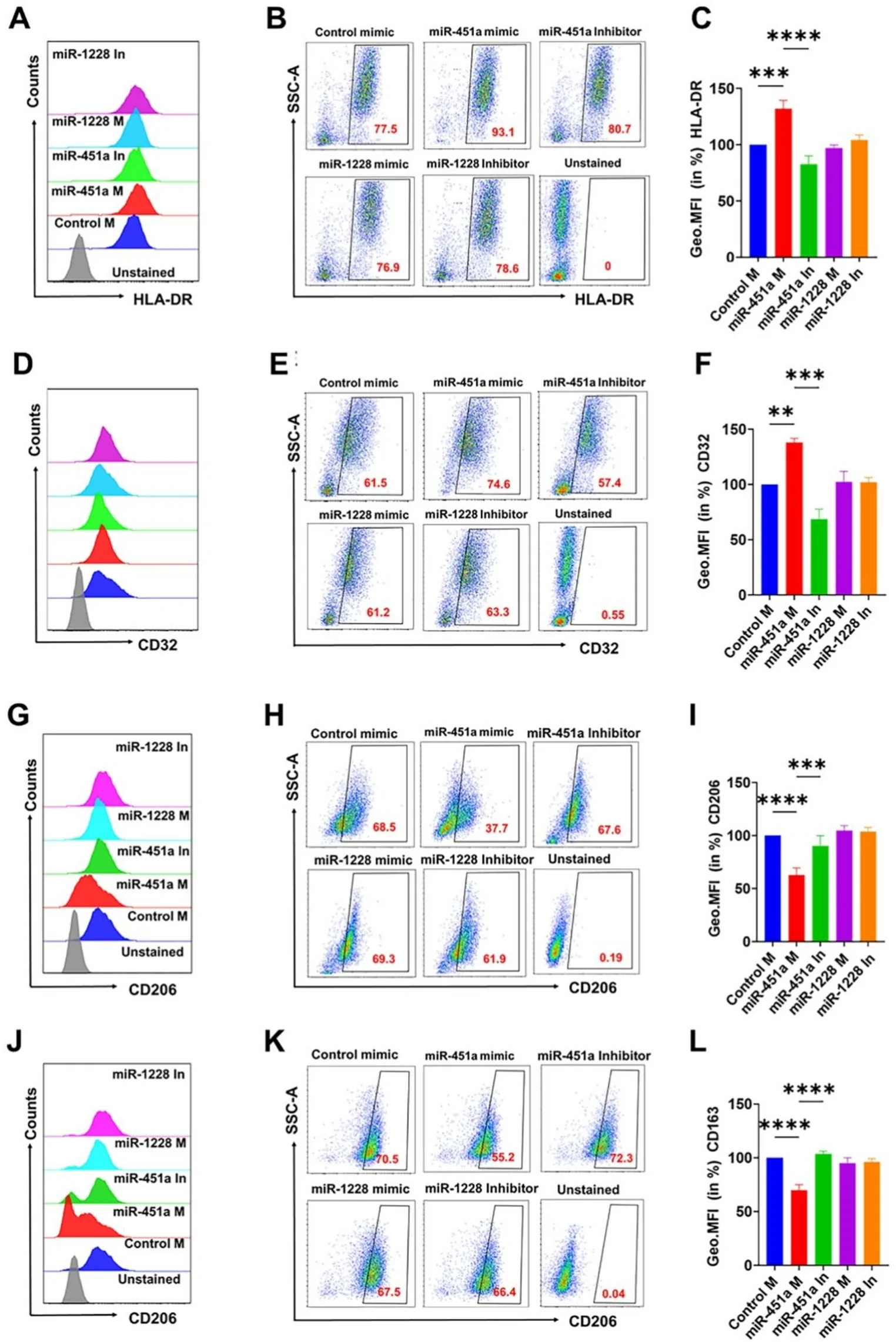
Overexpression of miR-451a impairs macrophage polarization by promoting M1 and suppressing M2 surface markers. Primary human M1- and M2-polarized macrophages were transfected on day 3 with miR-451a, miR-1228, or control miRNA mimics or inhibitors. After 72 h, cells were analyzed by flow cytometry for canonical M1 and M2 surface markers. Overlaid histograms show surface expression of the M1-associated markers **A** HLA-DR and **D** CD32, and the M2-associated markers **G** CD206 and **J** CD163 under each treatment condition. Representative scatter plots illustrate the proportions of cells positive for **B** HLA-DR, **E** CD32, **H** CD206, and **K** CD163, with percentages indicating the fraction of cells within each gated quadrant. Gating excluded debris and doublets, and analysis was restricted to viable cells within the main population gate. Normalized percent geometric mean fluorescence intensity (GeoMFI) for M1 and M2 markers in miR-451a-, miR-1228-, or control mimic–transfected cells is shown. GeoMFI values calculated relative to scramble control and expressed as percentages. Data is presented as mean ± SEM from four independent donors. Student’s t-test was used to calculate *p* values; statistical significance is indicated as **p < 0.01, ***p < 0.001, ****p < 0.0001

Primary CD14+ monocytes were transfected on day 3 of differentiation with miR-451a, miR-1228, control mimics, or inhibitors, and M1 or M2 surface markers analyzed by flow cytometry after 72 h. miR-451a overexpression, but not miR-1228, promoted M1 polarization while suppressing M2-like markers (n=6; Fig. 2A and D). Compared with control mimic, miR-451a increased the percentage of HLA-DR+ cells from 80.5 ± 3.15% to 91.3 ± 4.38% and CD32+ cells from 63.6 ± 4.62% to 79.2 ± 4.10% (Fig. 2B). In contrast, CD163+ cells decreased from 71.1 ± 2.38% to 36.4 ± 2.95%, and CD206+ cells from 67.1 ± 7.23% to 56.9 ± 3.44% (Fig. 2E). Normalized geometric mean fluorescence intensity showed similar changes, with increased HLA-DR (119.5 ± 6.36%) and CD32 (117.6 ± 2.25%) and reduced CD206 (70.3 ± 5.45%) and CD163 (81.4 ± 2.93%) (Fig. 2C and F). Overall, these findings suggest that miR-451a promotes a pro-inflammatory macrophage phenotype by increasing M1-associated markers while suppressing M2-associated markers.

### miR-451a directly binds to the 3' UTR and suppresses multiple M2 marker genes

**Fig. 3:**
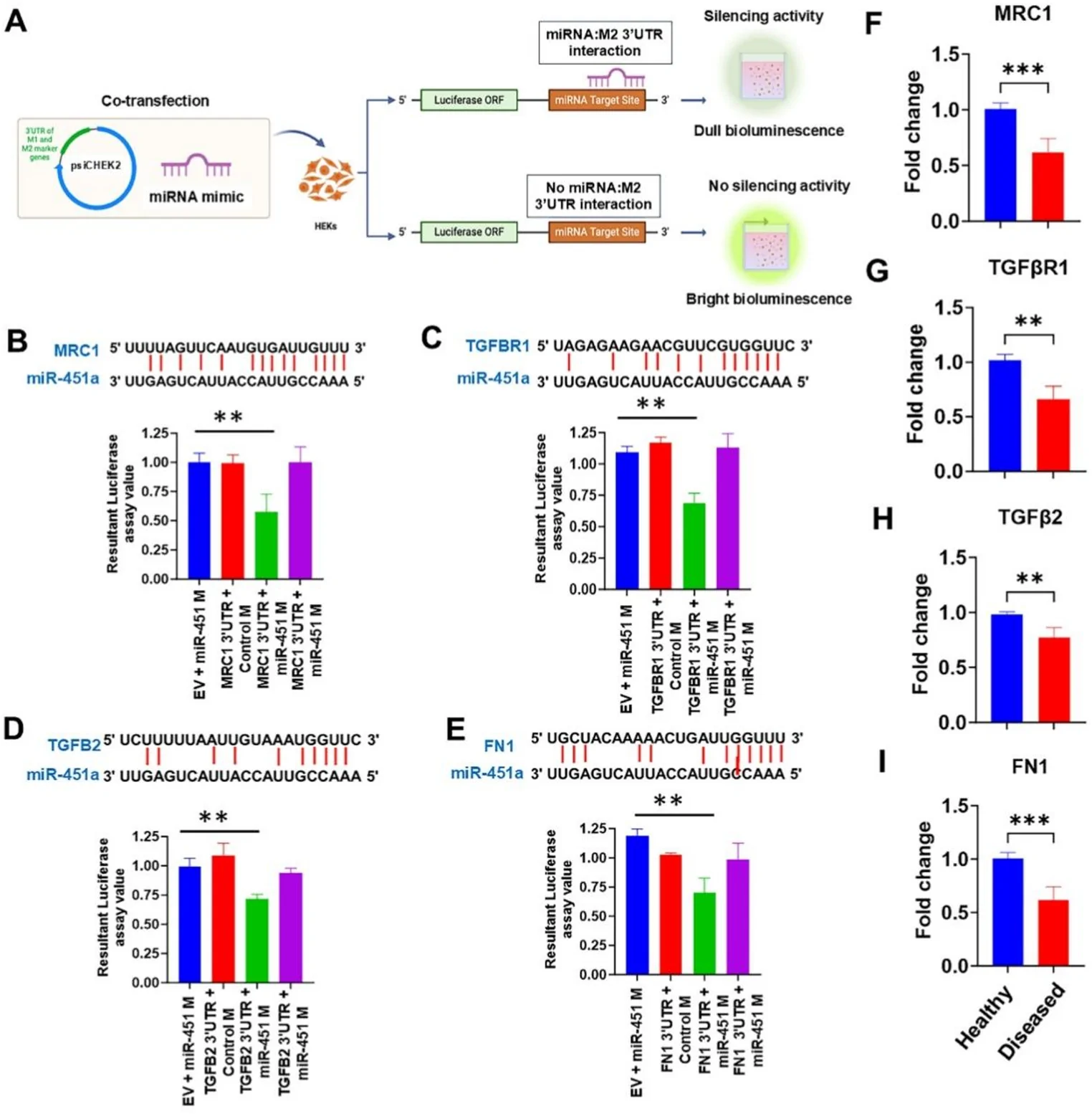
miR-451a-mediated repression of M2 macrophage markers correlates with altered expression in inflamed gingiva. **A** Schematic overview of the miRNA target-screening strategy using dual-luciferase reporter assays. Predicted miR-451a binding sites within the 3′UTR of **B** MRC1, (C) TGFBR1, **D** TGFB2, and **E** FN1 are shown as sequence alignments; only sites with minimum free energy (mfe) < −15 kcal/mol are displayed. HEK293 cells were co-transfected with dual-luciferase reporter plasmids containing the 3′UTR of MRC1, TGFBR1, TGFB2, FN1, or empty control vector, together with miR-451a mimic, control mimic, or miR-451a inhibitor. After 36 h, luciferase activities were measured, with Renilla activity normalized to firefly activity and further normalized to the empty vector + control mimic condition (set to 1). Data are presented as mean ± SEM from four independent transfections (*p < 0.05 versus control mimic; Student’s t-test). Total RNA was isolated from healthy (n = 8) and inflamed (n = 8) human gingival biopsies. Expression of validated miR-451a target genes **F** MRC1, **G** TGFBR1, **H** TGFB2, and **I** FN1 was quantified by RT-qPCR. Bar graphs depict relative gene expression in periodontally healthy versus inflamed gingiva. Statistical significance was evaluated by ANOVA (**p < 0.01, ***p < 0.001)

To determine whether miR-451a regulates M2-associated markers, we screened the 3′UTRs of MRC1, FN1, TGFB2, and TGFBR1 using RNA hybrid analysis. Putative miR-451a binding sites were identified in all four genes and cloned into reporter plasmids. A schematic of the dual-luciferase assay is shown in Fig. 3A, and sequence alignments with the 3′UTRs of MRC1/CD206, FN1, TGFB2, and TGFBR1 are presented in Fig. 3B–E (upper panels).

To validate these sites, HEK293 cells were co-transfected with psiCHECK2 alone or psiCHECK2 containing the cloned 3′UTRs together with miR-451a or control mimics. Compared with control mimic, miR-451a significantly reduced Renilla luciferase activity for MRC1, TGFB2, TGFBR1, and FN1 3′UTR constructs by ~30–50% (p < 0.05) (Fig. 3B–E, lower panels; n=4/group), identifying these genes as novel miR-451a targets linked to the M2 phenotype.

Consistent with these findings, gingival tissues from inflamed sites showed reduced MRC1 (0.68 ± 0.12 fold), FN1 (0.47 ± 0.07 fold), TGFBR1 (0.77 ± 0.09 fold), and TGFB2 (0.65 ± 0.12 fold) expression versus healthy controls (Fig. 3F–I). Together, these data support a model in which miR-451a suppresses M2-associated genes, limiting pro-resolving Mφ function in PD.

### miR-451a overexpression in macrophages attenuates bacterial phagocytosis

**Fig. 4:**
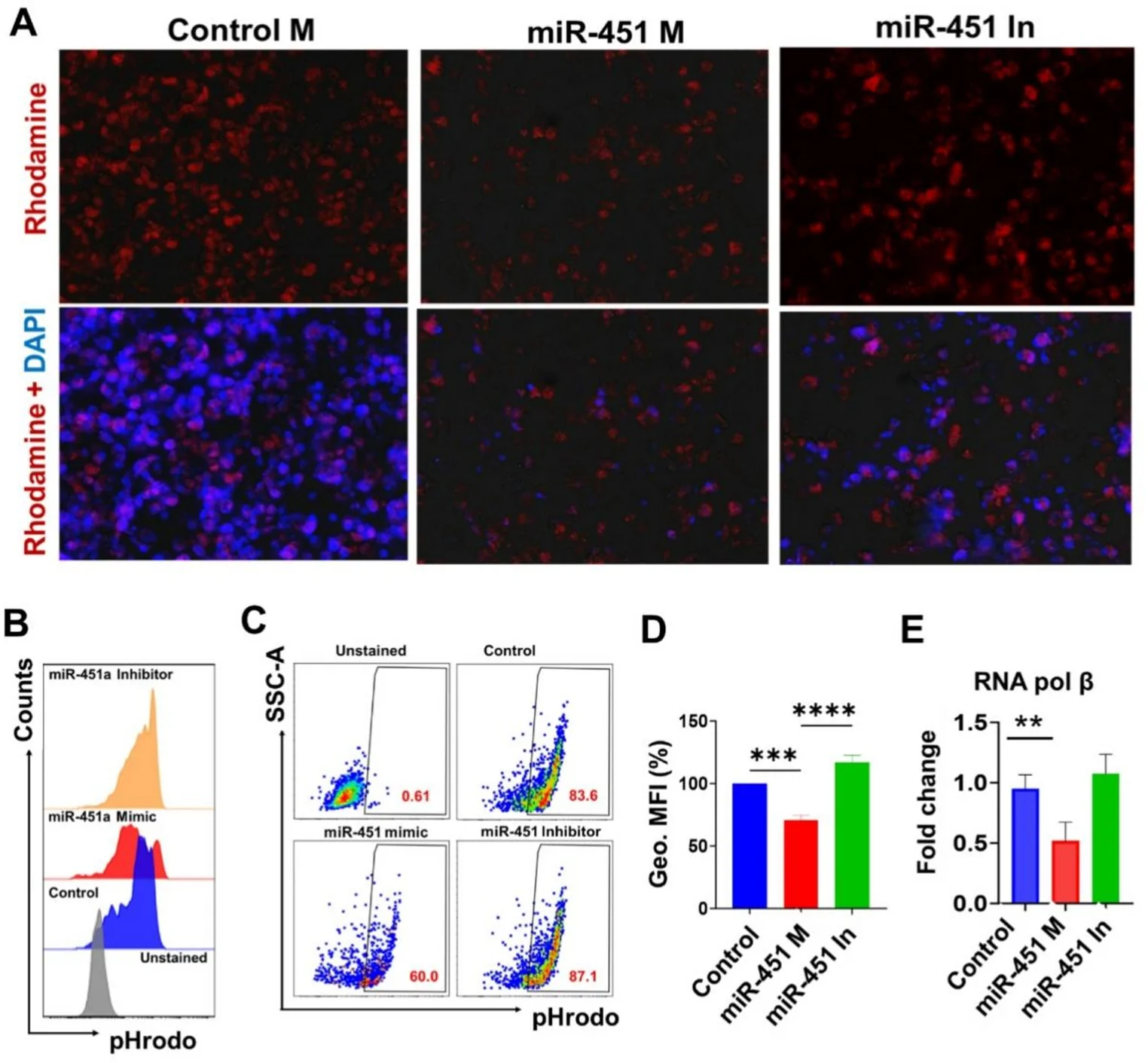
miR-451a overexpression attenuates bacterial phagocytosis by macrophages. M1-polarized human monocyte-derived macrophages (n = 4) were transfected with miR-451a or control miRNA mimics or inhibitors, and phagocytic activity was assessed using rhodamine-labeled *E. coli* bioparticles. **A** Representative fluorescence microscopy images showing bacterial uptake, visualized by rhodamine signal within macrophages (final magnification 40×; scale bar = 100 μm). **B** After 2–4 h of incubation with *E. coli* bioparticles, cells were harvested for flow cytometric analysis. Overlaid histograms demonstrate reduced rhodamine signal in miR-451a–transfected cells, indicating attenuated phagocytosis. **C** Representative scatter plots showing the percentage of Rhodamine⁺ cells, reflecting the proportion of macrophages that internalized rhodamine-labeled *E. coli*. **D** Bar graphs depict normalized percent geometric mean fluorescence intensity (GeoMFI) of rhodamine in macrophages, expressed relative to control-transfected cells. Macrophages were transfected with miR-451a mimic or control mimic and infected with *P. gingivalis* (100 MOI). Following phagocytosis assays, intracellular bacterial levels were quantified by qPCR analysis of the *P. gingivalis* RNA pol β gene. Bar graphs show relative *P. gingivalis* RNA pol β expression in miR-451a transfected cells normalized to control mimic, indicating bacterial uptake by macrophages. Statistical significance was evaluated by ANOVA (**p < 0.01, ***p < 0.001)

Macrophage phagocytosis of microbes and apoptotic cells is essential for periodontal tissue defense and homeostasis [36, 37]. To examine the role of miR-451a in Mφ function, M1 Mφ were transiently transfected with miR-451a mimic, control mimic, or inhibitor, and phagocytosis measured using rhodamine-labeled *E. coli* bioparticles. Fluorescence microscopy showed reduced *E. coli* uptake in miR-451a mimic-transfected cells compared with control mimic or inhibitor-treated cells (Fig. 4A). Flow cytometry confirmed this result, with *E. coli* uptake decreasing from 83.6 ± 5.15% in controls to 60.9 ± 5.5% in miR-451a-transfected cells (Fig. 4B, C; n=6/group). In contrast, miR-451a inhibitor-transfected cells showed uptake similar to controls (83.6 ± 3.2%) (Fig. 4C). Geometric mean fluorescence intensity of pHrodo+ cells was also reduced to 70.8 ± 3.89% (*P* < 0.0005) with miR-451a and increased to 117.0 ± 5.38% with inhibitor (Fig. 4D). These findings indicate that miR-451a attenuates bacterial phagocytosis by macrophages.

To further validate the role of miR-451a in regulating macrophage phagocytic activity, we examined its effect on the uptake of the periodontal pathogen *P. gingivalis*. Following macrophage transfection with miR-451a mimic or control mimic, cells were infected with *P. gingivalis*, and bacterial internalization was quantified by measuring intracellular bacterial DNA using pathogen-specific gene (RPL2) detection assays after phagocytosis. Consistent with our findings using *E. coli*, miR-451a overexpression significantly reduced macrophage uptake of *P. gingivalis* by ~60% compared with the control mimic, demonstrating attenuated phagocytic capacity in a clinically relevant periodontal pathogen (Fig. 4E; n=4/treatment). These findings further support a role for miR-451a in modulating macrophage antimicrobial functions and highlight its potential contribution to periodontal immune regulation.

### miR-451a potentiates inflammation by directly targeting SOCS3 and SOCS5

**Fig. 5:**
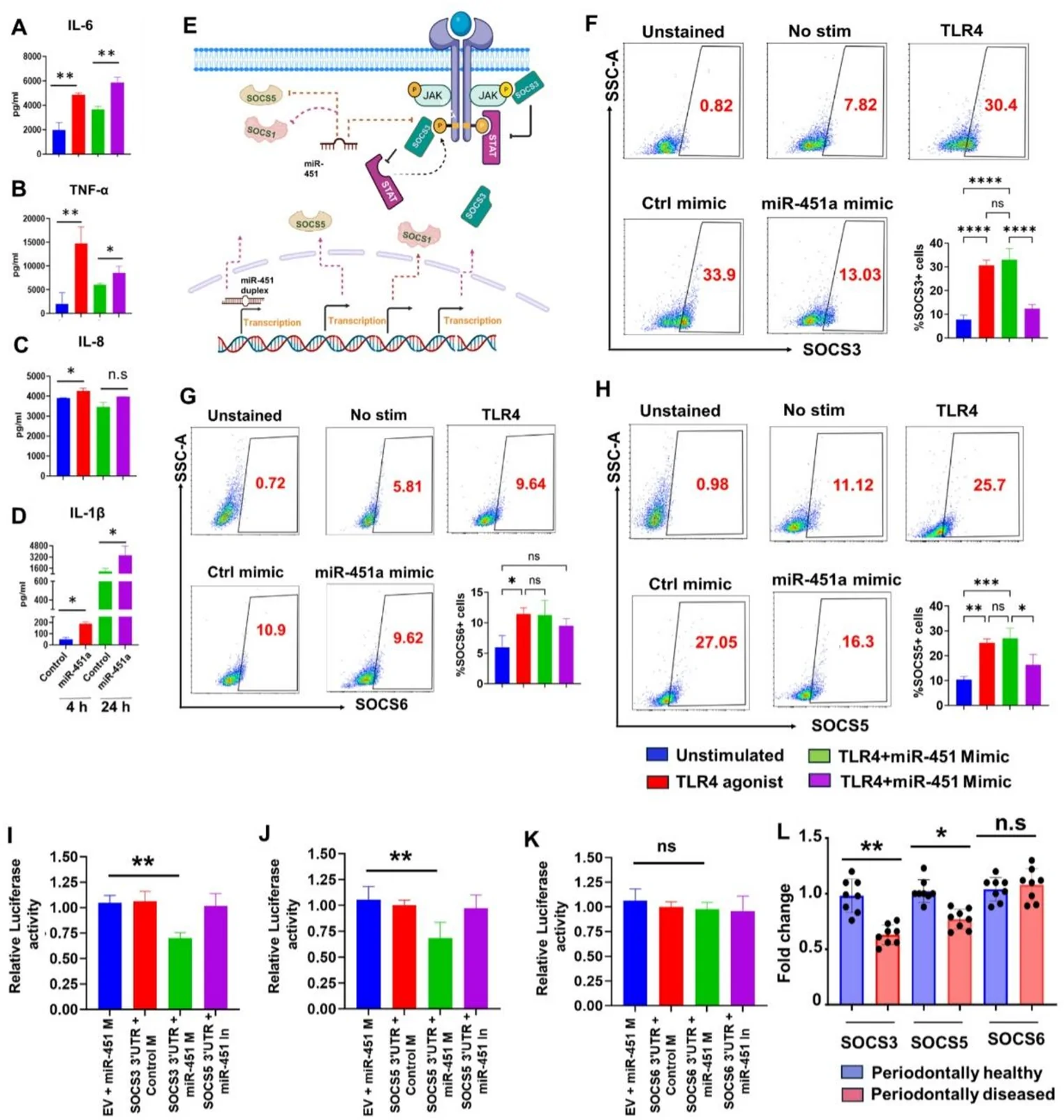
miR-451a potentiates pathogen-induced cytokine production by suppressing SOCS3 and SOCS5 expression in macrophages. Macrophages (n=4) were transfected with miR-451a or control mimic and after 36 h challenged with *E. coli* LPS (50 ng/mL). Culture supernatants were collected after 4 and 24 h and analyzed for secreted cytokines. Bar graphs show levels of **A** IL-6, **B** TNF-α, **C** IL-8, and **D** IL-1β. Data are presented as mean ± SEM from four independent donors. **E** Schematic summary of the anti-inflammatory role of SOCS proteins in the JAK-STAT pathway and the predicted miR-451a binding sites in SOCS3, SOCS5, and SOCS6. Expression of SOCS proteins were examined in macrophages (n = 4) transfected with miR-451a or control mimic. Upper panels show representative scatter plots of **F** SOCS3, **G** SOCS5, and **H** SOCS6 expression, with percentages indicating the proportion of positive cells within the indicated gates. Lower right panel **F**–**H**: Bar graphs showing normalized geometric mean fluorescence intensity (geo. MFI) values expressed as percentages relative to scramble control. Data is presented as mean ± SEM from four independent donors. HEK293 cells were co-transfected (n = 4) with dual-luciferase reporters containing the 3′UTRs of, **I** SOCS3, **J** SOCS5, **K** SOCS6 or an empty vector control, along with miR-451a mimic, or control mimic, or miR-451a inhibitor. After 36 h, luciferase activities were measured, with Renilla values normalized to firefly activity and further normalized to the empty vector + control mimic condition (set as 1). **L** Expression of SOCS3, SOCS5 and SOCS6 in healthy versus inflamed gingiva (n=8/group) was quantified by RT-qPCR. GAPDH was used as an endogenous control. Data are presented as mean ± SEM. Statistical significance was determined by ANOVA. *p < 0.05, **p < 0.01, ***p < 0.001

To determine whether miR-451a modulates inflammatory responses, Mφ were transfected with miR-451a mimic or control mimic and challenged with periodontal pathogens. Overexpression of miR-451a significantly enhanced pathogen-induced cytokine production. In response to *Pg*, miR-451a increased supernatant levels of IL-1β (2760.42 ± 257.30 vs 1711.36 ± 427.59 pg/mL), TNF-α (3632.52 ± 311.43 vs 2590.19 ± 326.47 pg/mL), IL-6 (2996.59 ± 752.69 vs 1800.22 ± 351.83 pg/mL), and CXCL8 (11,735.52 ± 1733.31 vs 8361.21 ± 1351.42 pg/mL) relative to control mimic (Fig. 5A–D). Similar effects were observed with *Aa* challenge, where miR-451a increased IL-1β (2505.08 ± 535.64 vs 1698.23 ± 328.40 pg/mL), TNF-α (3522.65 ± 680.64 vs 2537.30 ± 428.55 pg/mL), IL-6 (2700.33 ± 622.40 vs 1961.17 ± 472.09 pg/mL), and CXCL8 (8504.17 ± 1393.25 vs 6020.96 ± 282.33 pg/mL) (Fig. 5A–D). These findings indicate that miR-451a amplifies pathogen-induced pro-inflammatory cytokine production.

Pathway analysis identified immune signaling pathways as major miR-451a targets (Supplementary file 2). Because TLR signaling converges on the JAK-STAT pathway and SOCS proteins serve as key negative regulators, we examined the SOCS family for predicted miR-451a binding sites [38, 39]. SOCS3 contained one predicted site at 1854–1872, SOCS5 contained two predicted sites at 3282–3300 and 3302–3324, and SOCS6 contained two predicted sites at 2326–2343 and 2422–2439 (Supplementary files 2–8). No predicted binding sites were identified in the remaining SOCS genes. Figure 5E summarizes the anti-inflammatory role of SOCS proteins in JAK-STAT signaling and the predicted miR-451a target sites in SOCS3, SOCS5, and SOCS6.

To validate these predictions, Mφ were transfected with miR-451a or control mimic and then stimulated with the TLR4 agonist. SOCS expression was measured by flow cytometry. miR-451a significantly reduced the proportions of SOCS3-positive and SOCS5-positive Mφ compared with controls (Fig. 5F and G), while SOCS6 showed a modest but non-significant decrease (Fig. 5H). After TLR4 stimulation, the percentage of SOCS3-positive Mφ was lower in miR-451a-transfected cells than in controls (12.4 ± 1.7% vs. 33.02 ± 4.7%, *p* < 0.0001; Fig. 5F). A similar reduction was observed for SOCS5-positive Mφ (10.4 ± 4.2% vs. 28.1 ± 5.2%; Fig. 5G). In contrast, SOCS6 expression was not significantly altered (10.3 ± 1.02% vs. 11.3 ± 2.9%; Fig. 5H). As expected, TLR4 stimulation induced SOCS expression under control conditions. Together, these results show that miR-451a selectively suppresses SOCS3 and SOCS5 after TLR4 activation, supporting a role for miR-451a in limiting SOCS-mediated negative feedback within the JAK-STAT inflammatory pathway.

To investigate whether miR-451a directly regulates members of the SOCS family, HEK293 cells were co-transfected with psiCHECK2 alone or psiCHECK2 containing the cloned SOCS3, SOCS5, or SOCS6 3′UTRs together with either miR-451a or control mimics. Compared with the control mimic, miR-451a significantly reduced Renilla luciferase activity from reporters containing the wild-type SOCS3 (0.71±0.05; *p* < 0.01) and SOCS5 (0.68 ± 0.14; *p*<0.05) 3′UTRs, whereas no significant reduction was observed for the SOCS6 (0.97 ± 0.07; *p* > 0.05) 3′UTR construct (Fig. 5I–K). These findings demonstrate that SOCS3 and SOCS5, but not SOCS6, are direct targets of miR-451a.

Consistent with the reporter assay results, analysis of gingival tissues revealed significantly lower SOCS3 (0.63 ± 0.1; *p* < 0.05) and SOCS5 (0.74 ± 0.14; *p* < 0.05) expression in diseased tissues compared with periodontally healthy controls, whereas SOCS6 expression was not significantly altered (Fig. 5L). Importantly, the expression patterns of SOCS3 and SOCS5 were inversely correlated with miR-451a levels, supporting an antagonistic relationship between miR-451a expression and its target genes in vivo. Collectively, these findings provide direct mechanistic evidence that SOCS3 and SOCS5 are bona fide targets of miR-451a and support a model in which elevated levels of miR-451a suppress SOCS3/SOCS5 expression, thereby contributing to dysregulated cytokine signaling during periodontal inflammation.

## Discussion

Large-scale transcriptomic and miRNA profiling increasingly implicate post-transcriptional regulation in periodontal pathogenesis [40], but the contribution of non-canonical miRNAs remains poorly understood. In this study, we identify Dicer-independent miRNAs as potential regulators of periodontal immunity and tissue remodeling. Multiple non-canonical miRNAs, including miR-451a, miR-1228, miR-142-5p, miR-28-3p/5p, and miR-484, were markedly upregulated in inflamed gingiva, indicating that alternative miRNA maturation pathways are selectively engaged during disease. To our knowledge, this is the first evidence that non-canonical miRNAs are altered in PD and can polarize Mφ function. Similar stress-adaptive reprogramming of small RNA biogenesis has been reported in other inflamed tissues and may reflect the effects of microbial products, hypoxia, or inflammatory cytokines on miRNA processing [41, 42]. Non-canonical miRNA dysregulation is also documented in other inflammatory diseases. For example, miR-451a is elevated in systemic lupus erythematosus, Hashimoto thyroiditis, rheumatoid arthritis, sepsis, and Gram-positive bacterial infection, supporting its broader association with inflammatory activation [43–46]. Our findings suggest that bacterial virulence factors and oxidative stress in PD may similarly induce miR-451a and related non-canonical miRNAs.

Macrophages accumulate in periodontal lesions and acquire M1-like transcriptional features that enhance cytokine production and bone-resorptive pathways [47, 48]. Our earlier work linked miRNA dysregulation to oral mucosal immunity, including Mφ polarization and PD-associated noncoding RNA networks [6, 28, 29]. The present study extends those observations by identifying a non-canonical regulatory axis centered on miR-451a. We show that miR-451a is elevated in PD, correlates with M1 marker induction, and suppresses M2-associated markers in inflamed gingiva. Overexpression of miR-451a increased M1-associated markers HLA-DR and CD32, while reducing the M2-associated markers CD206 and CD163. This combined enhancement of M1 traits and repression of M2 features suggests that miR-451a polarizes Mφ toward a more inflammatory phenotype while limiting reparative programs. Mechanistically, miR-451a directly targeted several genes associated with M2 polarization and tissue repair. Dual-luciferase assays confirmed MRC1, FN1, TGFB2, and TGFBR1 as direct miR-451a targets. These genes are integral to Mφ alternative activation, matrix remodeling, and TGF-β signaling, all of which are important for resolution of inflammation and tissue repair. Consistent with these findings, expression of MRC1, FN1, TGFB2, and TGFBR1 was reduced in inflamed gingival tissues compared with healthy controls. These data support a model in which elevated miR-451a suppresses pro-resolving pathways in periodontal lesions, thereby sustaining inflammation and impairing its resolution.

Efficient phagocytosis is essential for microbial clearance and removal of apoptotic cells in periodontal tissues [37, 38]. We found that miR-451a overexpression significantly reduced uptake of labeled *E. coli* bioparticles, whereas inhibition of miR-451a had little effect. This indicates that increased miR-451a is sufficient to impair Mφ phagocytic capacity. In PD, such impairment could reduce bacterial clearance, prolong microbial persistence, and further amplify inflammatory signaling. Thus, miR-451a may contribute not only to inflammatory skewing but also to impaired innate immune function. Supporting this, exposure of macrophages to Pg and Aa induced miR-451a expression and enhanced inflammatory cytokine production, indicating that miR-451a is responsive to periodontal pathogens and may contribute to pathogen-induced inflammation. Functionally, miR-451a overexpression enhanced production of IL-1β, TNF-α, IL-6, and CXCL8. These findings suggest that miR-451a may establish a feed-forward loop in which bacterial stimulation induces miR-451a expression, thereby enhancing macrophage inflammatory activity. This mechanism may contribute to the persistence of inflammation in periodontal disease, where continuous exposure to bacterial stimuli promotes sustained immune activation and tissue damage.

To understand how miR-451a enhances inflammatory signaling, we examined its impact on the SOCS family, a key negative feedback system in cytokine and TLR signaling. Bioinformatic analysis identified predicted miR-451a binding sites in SOCS3, SOCS5, and SOCS6, and functional studies showed that miR-451a selectively reduced SOCS3 and SOCS5, but not SOCS6 expression after TLR4 stimulation. Because SOCS proteins negatively regulate JAK–STAT and related inflammatory pathways, suppression of SOCS3 and SOCS5 may prolong or enhance inflammatory signaling. Indeed, this mechanism is consistent with the observed increase in pathogen-induced cytokine production and suggests that miR-451a weakens endogenous anti-inflammatory checkpoints. Thus, miR-451a may function as a pro-inflammatory miRNA by promoting inflammation through suppression of endogenous inhibitory pathways. For instance, miR-155 promotes Mφ activation by targeting SOCS1 and SHIP1, has been implicated in inflammatory diseases including PD [49–54]. miR-21 enhances inflammatory signaling by targeting PDCD4, whereas miR-34a promotes obesity-associated inflammation by repressing Klf4 [54, 55]. Likewise, miR-30b and miR-181d aggravate myocardial inflammation through repression of SOCS3, relieving negative regulation of cytokine signaling [55]. Our findings suggest that miR-451a acts in a comparable way in PD. Specifically, miR-451a appears to promote gingival inflammation through two complementary mechanisms: suppression of M2-associated reparative programs and removal of SOCS-dependent inhibitory control over inflammatory signaling.

While this study provides novel mechanistic insights into the role of miR-451a in regulating periodontal inflammation, several limitations should be considered. First, clinical observations were derived from cross-sectional human gingival tissue analyses, and mechanistic studies were conducted primarily in vitro, limiting the ability to establish a causal relationship between miR-451a dysregulation and periodontal disease progression. Although SOCS3 and SOCS5 were identified as direct targets of miR-451a and exhibited an inverse expression pattern with miR-451a in diseased tissues, these interactions are considered a potential contributing mechanism rather than a definitively established causal pathway. Further mechanistic and clinical studies are needed to establish the causal role of miR-451 and SOCS3/5 axis in PD pathobiology. Second, the relatively modest clinical cohort size may affect the statistical power and generalizability of the findings. However, the split-mouth design, comparing healthy and diseased sites within the same individuals, minimizes inter-individual variability and strengthens the identification of disease-associated molecular changes. Larger, independent cohorts will be needed to further validate the clinical relevance of miR-451a as a potential biomarker or therapeutic target. Finally, while the M1/M2 macrophage polarization model is useful for characterizing inflammatory and reparative responses, it does not fully capture the complexity of macrophage heterogeneity in vivo. Future studies incorporating single-cell transcriptomics, spatial profiling, and in vivo models will help define how miR-451a regulates macrophage functional states and immune responses within the periodontal microenvironment.

In summary, this study identifies a distinct miRNA landscape in diseased gingiva enriched for Dicer-independent and other non-canonical species, with miR-451a emerging as a central regulator. Periodontal pathogen-responsive miR-451a promotes M1 Mφ polarization, suppresses M2-associated genes, reduces bacterial phagocytosis, and enhances inflammatory signaling through suppression of SOCS3 and SOCS5. These findings suggest that miR-451a may have potential as a biomarker and therapeutic target in PD, with possible implications for host-response modulation, microbial clearance, and tissue repair; however, further in vivo and clinical studies are needed to establish its diagnostic and therapeutic utility.

## Supplementary Information

Below is the link to the electronic supplementary material.

Supplementary Material 1

Supplementary Material 2

Supplementary Material 3

Supplementary Material 4

Supplementary Material 5

Supplementary Material 6

Supplementary Material 7

Supplementary Material 8

## Data Availability

The data that support the findings of this study are available from the corresponding author upon reasonable request.
